# Effectiveness of a multifactorial falls prevention program in community-dwelling older people when compared to usual care: study protocol for a randomised controlled trial (Prevquedas Brazil)

**DOI:** 10.1186/1471-2318-13-27

**Published:** 2013-03-15

**Authors:** Kelem de Negreiros Cabral, Monica Rodrigues Perracini, Aline Thomaz Soares, Francine de Cristo Stein, Celisa Tiemi Nakagawa Sera, Anne Tiedemann, Cathie Sherrington, Wilson Jacob Filho, Sérgio Márcio Pacheco Paschoal

**Affiliations:** 1 Department of Internal Medicine, Geriatrics Division, Hospital das Clínicas, Faculty of Medicine, Universidade de São Paulo, São Paulo, Brazil; 2 Geriatrics Discipline, Faculty of Medicine, Universidade de São Paulo, São Paulo, Brazil; 3 Master’s and Doctoral Programs in Physical Therapy, Universidade Cidade de São Paulo, Rua Cesareo Galeno, 448 Tatuapé, Sao Paulo 03071-000, Brazil; 4 Department of Physiotherapy, Speech-Language and Hearing Science and Occupational Therapy, Faculty of Medicine, Universidade de São Paulo, São Paulo, Brazil; 5 Musculoskeletal Division, The George Institute for Global Health, The University of Sydney, Sydney, Australia

**Keywords:** Accidental falls, Clinical trial, Aged, Exercise, Education

## Abstract

**Background:**

Falling in older age is a major public health concern due to its costly and disabling consequences. However very few randomised controlled trials (RCTs) have been conducted in developing countries, in which population ageing is expected to be particularly substantial in coming years. This article describes the design of an RCT to evaluate the effectiveness of a multifactorial falls prevention program in reducing the rate of falls in community-dwelling older people.

**Methods/design:**

Multicentre parallel-group RCT involving 612 community-dwelling men and women aged 60 years and over, who have fallen at least once in the previous year. Participants will be recruited in multiple settings in Sao Paulo, Brazil and will be randomly allocated to a control group or an intervention group. The usual care control group will undergo a fall risk factor assessment and be referred to their clinicians with the risk assessment report so that individual modifiable risk factors can be managed without any specific guidance. The intervention group will receive a 12-week *Multifactorial Falls Prevention Program* consisting of: an individualised medical management of modifiable risk factors, a group-based, supervised balance training exercise program plus an unsupervised home-based exercise program, an educational/behavioral intervention. Both groups will receive a leaflet containing general information about fall prevention strategies. Primary outcome measures will be the rate of falls and the proportion of fallers recorded by monthly falls diaries and telephone calls over a 12 month period. Secondary outcomes measures will include risk of falling, fall-related self-efficacy score, measures of balance, mobility and strength, fall-related health services use and independence with daily tasks. Data will be analysed using the intention-to-treat principle.The incidence of falls in the intervention and control groups will be calculated and compared using negative binomial regression analysis.

**Discussion:**

This study is the first trial to be conducted in Brazil to evaluate the effectiveness of an intervention to prevent falls. If proven to reduce falls this study has the potential to benefit older adults and assist health care practitioners and policy makers to implement and promote effective falls prevention interventions.

**Trial registration:**

ClinicalTrials.gov (NCT01698580)

## Background

The increase in non-communicable diseases (NCDs), including fall-related injuries, will be a major challenge in a progressively more globalized, urban and ageing world, especially in developing countries [1,2]. Falling in older age is a major public health concern due to the immediate injury-related consequences, such as fractures and traumatic brain injuries, but also due to the risk of associated long term disability, costly hospitalisations and subsequent nursing home admission [3,4]. One third of older adults aged 65 years and older fall at least once each year. This rate increases substantially with increasing age, and around 50% of people aged 80 years or more experience a fall event each year [5-7]. Brazilian cohort studies have shown similar rates [8,9].

Between 2% and 6% of falls result in fracture and the most common is hip fracture, [10] which is related with functional decline, death and increase in hospitalization costs [11]. Approximately 40% of total hospitalisations in the Brazilian public health system in 2009 were due to injury-related accidents [12]. A large cross-sectional study, using data from the Brazilian National Injury Surveillance System, revealed that among those aged 60 years and over emergency visits due to falls were associated with a reported disability [13].

Falls result from an interaction between environmental hazards and inadequate physiology to cope with the hazards, such as gait problems, poor vision, impaired peripheral sensation and lower limb strength, dizziness, and the use of psychotropic medication or polypharmacy [14,15]. Guidelines recommend a multifactorial fall risk assessment for all older adults who present for medical attention after a fall or who have gait or balance problems [16]. This assists with the identification of modifiable risk factors and with the implementation of targeted interventions for falls prevention [4]. The delivery of effective interventions for preventing falls in usual clinical practice presents a challenge for policy makers and health professionals [17,18].

A recent Cochrane review on falls prevention for older people living in the community included 111 randomised controlled trials (RCTs), with a total of 55,303 participants [19]. However, only four studies were conducted in developing, low-income countries [20-23]. The vast majority of RCTs have been conducted in developed countries, such as United States, Canada and Australia. This systematic review identified that multifactorial intervention, as well as exercise alone, either delivered as a multiple-component group exercise or home-based exercise, reduce fall rates but only exercise reduced risk of falls. Another systematic review has shown that exercise reduced the rate and risk of falling and there was a greater relative effect on fall rates observed in programs that included a combination of a higher dose of exercise (50 hours or more), progressive balance-challenging exercises and no walking program [24].

It has been recognised that multifactorial interventions that actively provide treatments aimed at reducing risk factors are more effective than those that provide referral and information alone [3,25]. Furthermore, existing trials of multifactorial programs are quite heterogeneous and different results in terms of fall rate reductions may be due to differences in participants’ sociocultural backgrounds, health care system characteristics and structures [19].

The aim of this study is to evaluate the efficacy of a multifactorial falls prevention intervention, composed of individualised risk factor management, exercise and educational/behavior change sessions, in reducing fall rates, in community-dwelling older adults living in Brazil when compared to usual care.

## Methods

### Trial design

**Figure 1:**
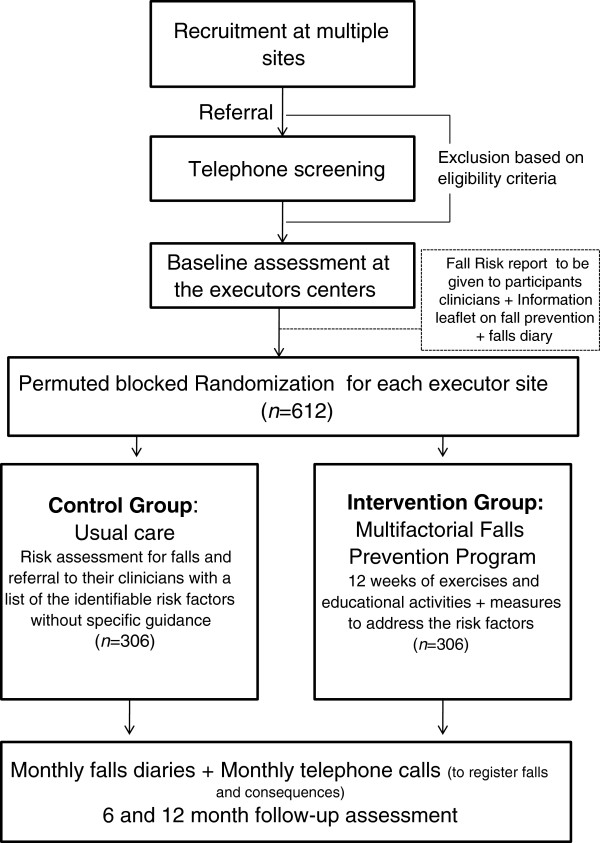
Design of study.

This study is a multicentre parallel-group RCT with 12 months follow-up (Figure 1). The Human Ethics Committee of the University of São Paulo Faculty of Medicine Clinics Hospital (CAPPesq 0145/11) has approved the trial and all participants will give written informed consent. Participants will be randomly allocated to either a “usual care” control group or the ‘multifactorial falls prevention program” intervention group. Eligible participants will be randomly allocated to study groups after the completion of a baseline assessment. The maximum estimated length between the baseline assessment and the randomisation will be 3 months.Group allocation will take place centrally using a computer-generated permuted block randomisation schedule by an investigator not otherwise involved in recruitment or data collection (i.e., a concealed randomisation system). Assessors will be blinded to group allocation but due to the nature of the trial, the participants will not be blinded to group allocation.

### Participants

Community-dwelling men and women aged 60 and over who have fallen at least once in the last 12 months will be included. A fall will be defined as “an unexpected event in which the participants come to rest on the ground, floor, or lower level.” Participants will be asked to answer the question: “In the past 12 months, have you had any fall including a slip or trip in which you lost your balance and landed on the floor or ground or lower level?” [26].

At initial contact potential participants will be screened by telephone to ensure they have fallen at least once in the past 12 months and will be able to attend the evaluation centre once a week for 12 weeks. If there is an affirmative answer to both questions, the person will be invited to attend the evaluation centre to be assessed for eligibility.

Participants will be excluded if they have:

• A previous diagnosis of dementia or a cognitive decline that prevents the understanding of simple instructions or guidelines;

• A previous stroke with a severe neurological impairment, such as loss of strength, and perceptual or language limitations;

• A progressive neurological disease (e.g. severe Parkinson’s disease);

• A severe visual deficiency;

• Any acute illness that the physician considers as an exercise contra-indication (e.g. uncontrolled angina, acute coronary disease);

• An acute vertigo or dizziness less than3 months duration;

• Inability to maintain a standing position, even with the use of a walking aid or other device.

Participants will also be excluded if they unable to communicate due to an aphasia, severe hearing loss even with the use of hearing aids or those who do not have mastery in Portuguese language. People will also be excluded from participation if they are engaged in a regular exercise program that is likely to challenge balance, including physical therapy, with a frequency of equal to or more than twice a week such as: muscle strengthening, balance or gait exercise, Tai Chi and Yoga. People will not be excluded if their regular exercise regime is limited to walking, water-based exercise or any other form of therapy that does not include the exercises described above (e.g., thermotherapy, spine and shoulder exercises, etc.).

### Setting and recruitment

Participants will be recruited in 4 referral centers in Sao Paulo, Brazil (Geriatrics Service at the University of São Paulo Faculty of Medicine Clinics Hospital; the Institute of Geriatrics and Gerontology “José Ermírio de Moraes”; the Reference Centre for the Elderly in the North Zone and the Paula Souza Primary Care Centre).

The Faculty of Medicine Clinics Hospital is a public complex of health institutions which offers 2,200 beds, distributed between its six specialized institutes and also outpatient services and rehabilitation. The Institute of Geriatrics and Gerontology is located in a very populated area, the east zone of Sao Paulo and is reference for 41 primary health units. The Reference Centre for the Elderly in the North Zone covers 142 primary health care units. The Paula Souza Primary Care Centre is located in the west zone of São Paulo.

The intervention will be delivered in 3 sites: Geriatrics Service at the University of São Paulo Faculty of Medicine Clinics Hospital; the Institute of Geriatrics and Gerontology “José Ermírio de Moraes”; the Reference Centre for the Elderly in the North Zone, where a multidisciplinary team will be trained to deliver the program. Figure 1 describes the study design.

### Control group

The control group will receive a baseline assessment to identify risk factors for falls and will be referred to their clinicians with a report of individual modifiable risk factors to be managed without any specific guidance: referral to routine services or specific interventions will be at the discretion of their primary clinicians. So, further management of each participant in the control group will be individualised with no specific protocol. Interventions will be recorded. Participants will receive a leaflet with general information about fall prevention and instructions to fill in a monthly fall diary. They will be telephoned each month to ask for the information regarding falls and their consequences.

### Intervention group

The intervention group will also receive a baseline assessment to identify risk factors for falls and will be referred to their clinicians with a report of individual modifiable risk factors so they will be able to receive usual care until they start to attend the Multifactorial Falls Prevention Program. Participants will also receive a leaflet with general information about fall prevention. The Multifactorial Falls Prevention Program will be a 12 week group-based intervention for 10 to 12 participants per group, with two and a half hour session per week. Once a month, there will be an additional one hour medical evaluation. During the intervention period participants will also keep their regular visits to their primary care clinicians. Clinicians of each participant in the intervention group will be contacted and encouraged to motivate their patients to adhere to the program, especially the home-based exercises.

The program will consist of:

1. A progressive, structured, *on-site exercise program* consisting of specific balance and strengthening exercises delivered by trained physiotherapists, who will undergo a day-long training session and receive a training manual. The group-based program will be conducted on a weekly basis, and will last for an hour and a half each session. In addition a *home-based exercise program* will be performed twice a week at home. The physiotherapists who will deliver the on-site group-based program will also teach the home-based exercises.

### On-site, supervised exercise program

The program will consist of progressive balance training, including postural orientation and anticipatory postural adjustments, sensory-motor activities, and strengthening exercises to enhance balance and postural control and reduce falls. Exercises in the standing position will be prioritised in order to target balance control. It has been found that programs that challenge balance and use a higher dose of exercises have greater relative effects on falls prevention [24]. In addition, exercises will be included to improve trunk flexibility, ankle mobility and proprioception. Exercises will be both static and dynamic, and will progressively increase in intensity to maximise strength and balance gains.

The group exercise protocol will comprise a fifteen minute warm-up followed by 45 minutes of a core set of balance and strengthening exercises performed in a standing position. The initial exercise dosage and level of difficulty will be individually prescribed by a physiotherapist after an assessment of each participant’s physical abilities. As gains are made, the exercises will be progressed in terms of number of repetitions to be completed, time to sustain a position, intensity of resistance used and task difficulty. For example, balance exercises will be progressed by using a narrower or unstable base of support, applying visual information deprivation, reducing upper limb support and using dual-task activities. The exercises will be demonstrated and supervised to ensure correct technique is used and the risk of falling is minimised. Initially participants will carry out the exercises next to a chair or to the wall, for immediate support in the event of loss of balance or if the participant feels insecure, however the use of upper limb support will be minimised when it is safe to do so to maximise the challenge to balance. The progression of exercises will also take into account the individual level of exertion required as measured by the Borg Rating of Perceived Exertion scale at the end of each exercise session.

The strengthening exercises will target ankle plantar flexors and dorsi flexors, knee extensors and hip abductors. Participants will use weight belts. Examples of the specific exercise to be included are heel raises in standing, forward and lateral step-ups onto a small block and sit to stand practice [27]. The balance exercises will consist of postural orientation exercises (weight bearing transfer in all directions of limits of stability, preferably using ankle motor strategy), static and dynamic balance exercises. The balance exercises will be included in a circuit protocol with different motor, sensory and cognitive tasks such as semi-tandem and tandem stance, stepping onto a block with alternate feet as quickly as possible, stepping and walking in different directions and at different velocities, negotiating obstacles and different surfaces, reaching for objects and making turns while walking. The complete exercise protocol will be available in a specific study manual and a training video.

### Home-based exercise program

Participants will be provided with a detailed booklet containing safety precautions, instructions and photographs of exercises for use in home exercise sessions. They will be instructed to do the exercises under the supervision of a relative or caregiver when possible, in a suitable place (with good lighting and ventilation) and using appropriate support (close to a table or chair, or the corner of a wall). At the first four group-based exercise sessions, additional instructions on how to carry out the home exercises will be provided to all intervention group participants. The exercises will be reviewed, if necessary, after each session until the last week of intervention. All necessary equipment to undertake the exercise program will be provided. Participants will be given an exercise diary for recording the frequency and number of repetitions of each exercise they perform as well as any minor adverse effects (e.g. muscle soreness, pain). The exercise diaries will be returned to the researchers at the 12 month re-assessment appointment. To avoid revealing study group allocation to the blinded assessor, exercise diaries will be placed in a locked box at the study site reception.

After a brief warm-up the home-based exercises will consist of similar exercises to those performed in the group sessions. Exercises will focus on strengthening of the ankle plantar flexors, knee extensors and hip abductors, performed in a standing position so that balance is challenged at the same time. Other exercises will include standing in a semi-tandem position, tandem walking and trunk righting. The progressions of home exercises will be concurrent with those done on-site in the group sessions. At the end of the intervention period, the participants will have reached at home the last progression level reached on-site and will be encouraged to maintain the dosage and progression level.

2. Educational/behavioural sessions addressing specific environmental and behavioural risk factors for falls will be delivered by trained health professionals. The 12 educational sessions will last for 40 minutes each and will use appropriate language for lay people. Audio-visual material will be used to support the verbal content when appropriate. Each meeting will consist of a brief introduction to the core topic for the day with supporting audio-visual or graphical material. This will be followed by group discussion to identify the beliefs, experiences and attitudes of participants regarding the discussion topic and any major barriers to the implementation of the suggested preventive strategies. In addition to discussing intervention strategies from the point of view of fall prevention, other benefits will be highlighted, such as for general health and improving or maintaining functional independence. The educational session will always end with a summary of the core messages of the session. Topics to be covered in the sessions are included in Table 1. The health care professional responsible for delivering the educational/behavioural sessions will attend a one-day training session and will receive a manual containing the specified core components of each session, learning strategies, goals to be achieved and messages to be delivered to participants.

3. A medical evaluation of modifiable risk factors not amenable to exercise will be performed by trained doctors, based on the currently available evidence [16]. The following risk factors will be addressed:

i. Postural hypotension

Participants with postural hypotension will be recommended to use pressure stockings and will receive counselling on associated behavioural factors, such as adequate fluid intake, sleeping with an elevated head, avoiding sudden changes in position and making circular movements with the feet before standing up. A review will be conducted into the use of medications that may contribute to postural hypotension such as antihypertensives, alpha agonists and tricyclic antidepressants and a contact with the primary care physician will be made to discuss any medication withdrawal. In regards to postprandial hypotension, participants will be encouraged to eat small, frequent meals, and to lie down after each meal.

ii. Visual impairment

Participants identified as having visual problems will be referred to an ophthalmologist. They will also be oriented in the proper use of bifocal or multifocal lenses while walking on the street and using stairs or ramps.

iii. The use of four or more medications or the use of psychotropic medication

A review of the risk-benefit of using specific medications will be undertaken, especially psychotropic and anti-hypertensive drugs and a contact with the primary care physician will be made to discuss any medication withdrawal. Counselling on avoiding self-medication will be provided.

iv. Undernutrition

Participants with a BMI lower than 22 kg/ m2 and/or a leg calf circumference under 31 cm will be instructed to increase their protein and calorie intake.

**Table 1:** Educational topics covered during intervention

**Week no.**	**Session**
Week 1	Introduction to the Program for Prevention of Falls.
Week 2	Why do the elderly fall? Is falling normal in old age?
Week 3	The importance of exercise in strengthening muscles, improving balance and walking safely.
Week 4	What is risky behaviour? Is it normal to be afraid of falling? Which actions do I need to modify or avoid preventing falls?
Week 5	Medication and falls
Week 6	Is my home safe? How can I make it safer?
Week 7	How can I care for my feet? What is a safe shoe?
Week 8	Does food increase my risk of falling? How can I eat well?
Week 9	How good is my memory and attention span? What can I do to improve them?
Week 10	Do I see well? How important is my vision for safe indoor and outdoor mobility?
Week 11	Osteoporosis and the risk of fracture.
	How to get up from the floor after a fall.
Week 12	Why do I need to continue exercising?

### Outcomes

#### Data collection

Data will be collected from the baseline and follow-up physical assessments, monthly falls diaries, telephone contacts and medical and expert reports. The assessments will be conducted by independent, trained health care professionals who will not be providing the interventions and that will be unaware of the participants study group allocation. Participants will be instructed not to inform the assessors of their intervention status. Assessors will complete a questionnaire to assess their perception of the participants’ group allocation, in order to test the success of assessor blinding. The assessments will include questionnaires, physical examination and physical performance tests. All participants will undergo three assessments: the first at baseline prior to randomisation, the second and third at 6 and 12 months after commencement of the intervention respectively.

#### Primary outcome measures

The primary outcome will be the rate of falls and the proportion of fallers in 12 months after randomisation. Participants will receive instructions to fill in a monthly fall diary. They will receive telephone calls each month to ask for information regarding falls and their consequences, such as the mechanisms, environmental conditions, location (indoors or outdoors), activity during the fall and injuries sustained.

#### Secondary outcome measures

Secondary outcome measures will be the risk of falling, fall-related self-efficacy score, measures of balance, mobility and strength, health services use and difficulty with daily tasks. These outcome measures are described below.

**Fall-related self-efficacy** will be assessed with the *Falls Efficacy Scale International*[28], translated and adapted in its Brazilian version [29], which consists of a questionnaire with16 activities in which the participant rates, on a 4-point scale their degree of concern about the possibility of falling while carrying out the activities. It measures their degree of self-efficacy to avoid falling.

**Balance mobility and strength** will be measured by the: *Berg Balance Scale*[30-32], *Alternate Step test*[31], *Sit to stand Test*[33,34]*and Hand grip strength*[35,36]. The Berg Balance Scale consists of 14 common tasks involving static and dynamic balance, such as reaching, turning, moving, standing and getting up. Ability to perform the tasks is graded from 0 to 4, with an overall maximum score possible of 56 points, and higher scores indicating better balance. The *Alternate Step test*[31] requires the participant to stand in front of an 18-cm high step or stool and at a verbal command the participant taps the whole foot (shoes removed) onto the step, and alternates with the right and left feet, for a total of eight repetitions as quickly as possible. The time taken to complete the task is the score. The *Sit-to-stand test* will be used to primarily assess lower limb strength. There will be a pre-test which involves the patient moving from sitting in a chair to a standing position without the assistance of their hands. If the subject can perform this movement, they will be instructed to repeat the test, as quickly as possible, five times consecutively with their upper arms crossed over their chest. The time taken to perform this task will be recorded. *Hand Grip strength* will be assessed using a portable hand dynamometer (SAEHAN® model SH5001). The participant will be seated with their shoulder in a neutral position and their elbow flexed at 90°.Three attempts will be performed alternately in each hand; the mean of the three measures will be recorded.

**Fall risk** will be assessed using the *QuickScreen Clinical Falls Risk Assessment*[37], a validated instrument consisting of five physical performance items, two questions about medications and one about previous falls. An individual’s estimated fall risk will be considered to have decreased if, upon retest, they have moved down to the next level in the risk categories proposed by Tiedemann et al. [37]. The risk categories and their associated probabilities of future falls are as follows: for 0–1 QuickScreen items, the probability of falling is 7%, for 2–3 items the probability of falling is 13%, for 4–5 items the probability of falling is 27% and for 6 or more items the probability of falling is 49%.

**The level of *difficulty with daily tasks*** will be assessed using the Brazilian OARS Multidimensional Functional Assessment Questionnaire (BOMFAQ) adapted from the Older Americans Resources and Services (OARS) [38] instrument that measures self-reported difficulty while performing 15 daily activity tasks: eight activities of daily living : lying down and getting up from bed, eating, combing hair, dressing, bathing, walking on a uneven surface, getting to the toilet in time, cutting toenails and seven instrumental activities of daily living: taking medications, climbing stairs, walking near home, preparing meals, taking public transport, shopping and cleaning the house, hierarchically organized. The number of activities performed with difficulty will be summed and categorized as follows: 0, 1 to 3, 4–6 or 7 or more activities.

**Services use** will be measured by the number of fall-related visits to emergency departments and hospitalisations (frequency and duration) per participant.

**Compliance** with the home exercise program (frequency and duration) will be recorded in a home exercise diary. Adverse side effects will be measured with an adverse events form, including stiffness, pain, fatigue, etc. At the end of the follow-up period participants will be asked to identify reasons for non-adherence on the home-based exercise program.

### Baseline assessment of clinical fall risk factors

Individual clinical fall risk factors will be established through questions about urinary incontinence, diabetes, alcohol consumption, physical activity level, medication use and the number of hospitalisations or emergency department visits during the past year. Physical examination will be undertaken by trained physicians and will consist of:

*Postural hypotension* identified by assessment of blood pressure after 5 minutes in the supine position and after 2 minutes in the standing position. Abnormality is defined as a difference of 20 mmHg or a 20% decrease in systolic BP with or without symptoms, or a reduction in the diastolic BP of 10 mmHg. *Vibratory perception* will be assessed using a vibrating tuning fork of 128Hz applied over the bony part of the dorsal distal phalanx of the hallux*. Cognition* will be assessed with the Mini Mental State Examination (MMSE) [39]. *Depressive Symptoms* will be assessed by the Geriatric Depression Scale (GDS) translated and validated in Brazil. The short version has 15 questions with a yes/no response concerning the participant’s emotional state in the week preceding the assessment. A score of 6 or greater indicates a depressed mood [40,41]. *Visual acuity* will be measured using a directional Table E or Snellen (Snellen-E chart), which is a standardised measure of vision, conducted at a distance of five metres. As the intention is to measure functional vision, the participants will be allowed to wear their usual corrective lenses, and both eyes will be evaluated simultaneously. Values below 0.3 will be considered to indicate impaired vision. *Nutritional Status* will be measured with Body Mass Index (BMI) and calf circumference. The following BMI classifications will be used: BMI < 22 kg/ m^2^ = underweight, between 22 and 27 kg/ m^2^ = normal weight, >27 kg/ m^2^ = obese [42].

### Sample size calculation

A total of 612 participants (306 per group) will provide 80% power to detect as significant, at the 5% level, a 30% lower rate of falls (i.e. the primary outcome measure) for intervention group participants compared with control participants (i.e., IRR = 0.70). For the sample size calculation we used the gennbreg command in Stata and coefficients from previous studies: alpha (a measure of over-dispersion in the negative binomial regression model) was assumed to be 0.76 based on data from a previous trial [43]. We assumed the control group rate of falls would be 1 fall per person over the 12-month follow-up period. An average follow-up period of 11 months (rather than the planned 12 months) was used in these calculations to account for loss to follow-up.

### Statistical analysis

The primary analyses will be conducted using the intention-to-treat principle. Fall data will be analysed using all randomised participants until they have died or have withdrawn from the study. Demographic and clinical characteristics at baseline will be summarized with descriptive statistics. The incidence of falls with 95% confidence intervals (CIs) in the two groups (intervention group and control group) will be calculated and compared using negative binomial regression analysis. The proportion of fallers in the two groups will be compared with the relative risk statistic. General linear models will be used to assess the effect of group allocation on the continuously-scored secondary outcome measures after adjusting for baseline scores. Logistic regression models will be used to compare groups on dichotomous outcome measures. Interactions between subgroup variables (past falls and mobility-related disability) and group allocation will be tested, and in the case of a significant interaction, the comparison between the intervention group and control group will be reported within the subgroups. Statistical analyses will be undertaken using the SPSS and Stata software packages. P < .05 will be considered statistically significant.

## Discussion

There is a substantial amount of evidence that falls in older adults can be reduced by different interventions in multiple settings. However, interventions in developing countries with limited resources are scarce and yet to be fully investigated.

Thus, we aim to develop a program which is feasible in terms of intensity and characteristics, combining specific on-site approaches during a short period of time with home-based exercises. We believe that these strategies, if proved to be effective, could be implemented by a small team of primary health care professionals with a small amount of training.

This study will also help to understand home-based exercise adherence predictors in this population. The proposed sub-group analyses may also identify specific participant groups that are most likely to gain benefit from the intervention. Furthermore, secondary outcomes analyses will determine if there is any significant improvement in physical functioning and disability in older people as a result of the intervention, which to our knowledge has not been investigated previously with older Brazilian adults over a 12 month follow-up period.

In summary, this study is the first randomised controlled trial of a multifactorial falls prevention program to be conducted among Brazilian older adults. The pragmatic design of the intervention will ensure that study results can be implemented into clinical practice with minimal effort. If proven effective, this fall prevention program will benefit older adults and assist health care practitioners and policy makers to make better choices concerning falls prevention interventions.

### Trial status

The trial is currently in the recruitment phase.

## Competing interests

The authors declare that they have no competing interests.

## Authors’ contributions

This manuscript was drafted by MRP, KCC,FCS and AT. CS contributed to the study design and sample size calculation. KCC, FCS, ATS, MRP, SMPP and WJF were responsible for the grant application for this project. All other authors contributed to its critical review and approving the final draft. All authors read and approved the final manuscript.

## Pre-publication history

The pre-publication history for this paper can be accessed here:

http://www.biomedcentral.com/1471-2318/13/27/prepub
